# Intubation in Specific Stenosis of the Larynx

**Published:** 1891-07

**Authors:** John O. Roe

**Affiliations:** 28 North Clinton Street; Rochester, N. Y.


### INTUBATION IN SPECIFIC STENOSIS OF THE LARYNX.

By JOHN O. ROE, M. D., Rochester, N. Y.

In the afternoon of the second of last February, I was hastily sent for by Dr. Rutherford, of Rochester, to see, with him, a patient who was suffering from great dyspnea.

I found on my arrival a woman about forty years of age, who was so much oppressed for breath that she was quite cyanotic. Hastily examining her larynx I found it almost entirely occluded by a subglottic swelling, projecting from the left side of the larynx and pressing the left vocal cord upwards so as to give it quite a bulging appearance.

The swelling was quite firm, and from its appearance unquestionably a gummata.

This diagnosis was supported by the history of the patient and the presence of active syphilitic manifestations on other portions of the body at that time.

In order to avoid the operation of tracheotomy, I endeavored to cut away enough of the growth to give a free breathing space, but this could not readily be done without lacerating the left vocal cord, and I resorted to the expedient of introducing an intubation tube of an adult female size. The tube was of metal with hard-rubber head.

In order to introduce the tube past the growth, I had to use considerable force, but when the tube was in situ the dyspnea at once disappeared, and breathing was carried on through the tube with perfect freedom.

The patient got on admirably. She had no discomfort from the tube after the first day, when the sensation of pressure disappeared and deglutition even was very little interfered with by the presence of the tube.

The history of the case previous to the time I saw the patient is as follows:

About three years ago she was inoculated with a specific infection. This was soon after followed by an eruption and a conjunctivitis of the left eye; and afterwards at frequent intervals small patches of eruption would appear on various portions of the body, arms, legs, neck, face, etc., but confined mainly to the left side.

These small patches of eruption of a rupia nature would break down, suppurate, and after a time heal, leaving a scar, while other ulcers

1. Read before the Central and Western New York Medical Association, held in Buffalo, N. Y., June 2, 1891.

formed at different places in the same manner. When I saw her, there was one just above the left eye, one on the left shoulder and one on the left hip, which were all discharging quite profusely.

In February, 1890, during the first appearance of La Grippe, she began to be hoarse and to have some difficulty in breathing, which she attributed to an attack of the epidemic.

The trouble in breathing, however, slowly increased and by the last of December she began to have attacks of extreme dyspnea, particularly during the night, and for two or three days before I saw her she had become so choked that she was thought several times to be dying.

She was up to the time that Dr. Rutherford was called in (who at once recognized the formidable character of the laryngeal obstruction), in the hands of an irregular practitioner, who, it seems, knew the nature of the disease, but not enough to master it.

The patient was put on eighty grains of iodide of potassium daily, in conjunction with iodide of mercury. She improved rapidly, and in four days the tube was so loose that she coughed it out. Replacing it was not necessary, as the growth was very much reduced in size, owing to the combined influence of the pressure of the tube and the constitutional effect of the medicine. Every second day, applications of nitrate of silver, forty grains to the ounce, were made to the growth to stimulate and hasten its absorption, and at the end of four weeks it had nearly disappeared.

When I first saw the patient she could scarcely produce an articulate sound ; at the end of four weeks her voice had become a hoarse whisper and at the present time little hoarseness remains. There is some inflammatory thickening about the larynx, but the gummy growth has entirely disappeared.

The foregoing case is one of more than passing interest, since it illustrates so clearly the brilliant results that attend intubation of the larynx for obstructions due to chronic hypernia plasia and new formations, as well as for stenosis of the larynx of an acute inflammatory nature.

Thus far I have found records of ten cases in which intubation has been employed for syphilitic obstructions of the larynx. Two of them were for acute syphilitic stenosis of the larynx, in which condylomata and gummata were present ; two for chronic syphilitic stenosis of the larynx, with hypertrophy and ulceration ; three for cicatricial stenosis of the larynx, following ulceration ; two for chronic syphilitic stenosis of the larynx, with bilateral paralysis of the abductors, in one of which there was also cicatricial adhesion of the vocal cords, and one for acute syphilitic stenosis of the larynx, with perichondritis and anchylosis of the arytenoids.

In all these cases, as in the case I have just reported, complete relief and cure followed the introduction of the tube.

The advantages of this method are: The operation is quickly done; it is quite free from danger; it avoids the formidable operation of tracheotomy with all its attending sequelæ, and it is the most efficient method of dilatation.

Previously, the only method of any value whatever for dilatation of cicatricial stenosis of the larynx was that of Prof. Shrœtter, and this was not always satisfactory, and, besides, required tracheotomy for the introduction of the dilator.

28 North Clinton Street.

